# German-style board games in the mental development of children

**DOI:** 10.1192/j.eurpsy.2022.1093

**Published:** 2022-09-01

**Authors:** A. Konovalova, A. Gasimov, K. Maslova

**Affiliations:** 1 I.M. Sechenov First Moscow State Medical University, Department Of Pedagogy And Medical Psychology, Moscow, Russian Federation; 2 Lomonosov Moscow State University, Faculty Of Psychology, Moscow, Russian Federation

**Keywords:** child mental development, factors of development, play activity, board games

## Abstract

**Introduction:**

Play activity has been studied from a scientific point of view relatively recently. Until the middle of the twentieth century, any games were considered only as a way of leisure and/or a tool for transmitting cultural experiences.

**Objectives:**

The research is aimed at studying play activity as a factor of mental development of a child.

**Methods:**

The method of work is a bibliographic analysis.

**Results:**

In psychology, the interest in the role of games in the psychological development of a child is primarily associated with the works of Z. Freud, J. Piaget, L.S. Vygotsky, D.B. Elkonin, who showed the importance of children’s imitation games: role-playing, directing, event-based (classification of E.O. Smirnova).

Since the 90s of the XX century, this hobby is becoming ever more common. At first, modern board games were created by adults for adults, and then there appeared board games specially designed for adults to play with children (family games) and for playing children’s groups.

Most of the board games popular with parents belong to the German school. Such games are characterized by relatively simple rules, a short or medium duration of the game, no direct confrontation between players and a low randomness in the course of the game (for example, Carcassonne, Catan, Ticket to Ride, etc.).

**Conclusions:**

German-style board games develop children’s communication skills, voluntary activity, abstract and formal-logical thinking, symbolic function, attention, the ability to cooperate (in cooperative games), imagination, and many games develop the child’s outlook and enrich the ideas about the world around and options for social interaction.

**Disclosure:**

No significant relationships.

